# Liver disorders and phytotherapy

**DOI:** 10.1016/j.toxrep.2025.102047

**Published:** 2025-05-10

**Authors:** Syed Sanober Qadri, Darakhshan Javaid, Adfar Reyaz, Shahid Yousuf Ganie, Mohd Salim Reshi

**Affiliations:** Toxicology and Pharmacology Laboratory, Department of Zoology, School of Biosciences and Biotechnology, Baba Ghulam Shah Badshah University, Rajouri, Jammu and Kashmir 185234, India

**Keywords:** Hepatotoxicity, Plant metabolites, Oxidative stress, Xenobiotics, Antioxidants

## Abstract

The liver is an essential organ crucial for metabolism, detoxification, and maintaining homeostasis, faces growing global health challenges such as alcoholic liver disease (ALD), non-alcoholic fatty liver disease (NAFLD), hepatitis, cirrhosis, and liver cancer. These conditions collectively account for significant morbidity and mortality worldwide. Although traditional treatments help control symptoms and slow disease progression, they are frequently hindered by issues such as drug resistance, side effects, and high costs, especially in areas with limited resources. Drug-induced liver injury (DILI) continues to be a significant concern. Traditional medicine offers a promising avenue for addressing these limitations, with numerous plants demonstrating hepatoprotective properties through their bioactive compounds, including alkaloids, glycosides, and flavonoids. These natural agents not only mitigate hepatic damage but also provide immune modulation and chronic disease management. This review examines liver injury mechanisms and highlights the therapeutic potential of traditionally used medicinal plants in treating and preventing the liver diseases, emphasizing the integration of traditional knowledge with modern pharmacological advancements.

## Highlights

• Explores the etiology, progression, and global burden of chronic liver disorders, including hepatitis, NAFLD, and cirrhosis.
• Discusses the therapeutic potential of plant metabolites in mitigating liver disorders through various molecular mechanisms.
• Highlights antioxidant, anti-inflammatory, and hepatoprotective properties of phytochemicals in restoring liver health.
• Reviews bioactive compounds such as flavonoids, polyphenols, alkaloids, and terpenoids for their hepatoprotective efficacy.

## Graphical Abstract

**Figure:**
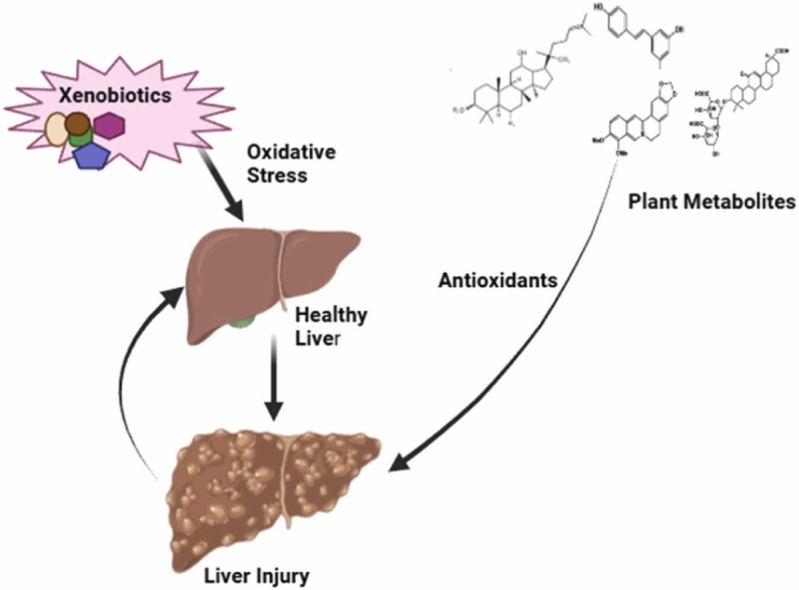

## 1 Introduction

The liver is an important organ as it plays a pivotal role in various essential functions, such as regulating macronutrient metabolism, supporting the immune system, maintaining blood volume, managing lipid and cholesterol balance, facilitating endocrine signaling, and detoxifying xenobiotic substances, including numerous drugs(Ullah et al., 2023). It is crucial in protein and amino acid metabolism. It metabolizes majority of proteins present in bloodstream, processes amino acids to generate energy and eliminates nitrogenous waste via urea metabolism[153]. Additionally, the liver detoxifies xenobiotics via biotransformation, an essential metabolic pathway for handling foreign substances [126].

Globally, liver diseases are emerging as a significant health concern, causing around 2 million deaths per year (One million deaths are attributed to complications of cirrhosis, with an additional half a million resulting from liver cancer and viral hepatitis [116]. Cirrhosis ranks 11th and liver cancer 16th in terms of mortality, collectively accounting for about 3.5 % of total global deaths [61]. The liver is highly prone to injury caused by viruses, alcohol, toxins and metabolic disorders, leading to an increased prevalence of hepatic conditions such as alcoholic liver disease (ALD), non-alcoholic fatty liver disease (NAFLD) hepatitis, and cirrhosis globally. Both NAFLD and ALD are characterized by excessive fat accumulation in the liver, though they arise from different underlying causes. ALD is caused by alcohol consumption with NAFLD commonly associated with metabolic syndrome. Nonalcoholic steatohepatitis (NASH), a more severe form of NAFLD, is characterized by inflammation and liver cell damage. It is closely associated with lifestyle factors such as obesity, insulin resistance, and metabolic syndrome. If left untreated, NASH can progress to more serious liver conditions, including fibrosis, cirrhosis, and potentially liver cancer, which may result in hepatocellular carcinoma and hepatic complications like ascites, encephalopathy, and variceal bleeding, leading causes of death globally [167].

Traditional medicine, with a long history across cultures, has played a crucial role in managing hepatic and other diseases. Natural compounds isolated from plants such as alkaloids, flavonoids and glycosides have shown notable pharmacologic effects, laying the groundwork for modern drug development. Increasingly, researchers seek to identify new hepatoprotective agents from plants to create innovative therapies for liver disorders [84]. While conventional treatments for liver diseases are effective in managing symptoms and slowing disease progression, they often come with limitations such as drug resistance, side effects, and, in some cases, drug-induced liver injury (DILI), where medications paradoxically damage the liver [89]. The high cost and need for lifelong treatment also add to the burden, especially in low-income regions, while the lack of personalized approaches limits efficacy for some patients. This review highlights the bioactivity of numerous plants, identifying their bioactive compounds and elucidating the mechanisms through which they combat hepatotoxicity. This evidence supports integrating traditional medicinal knowledge with contemporary approaches, especially for hepatoprotection, immune modulation, and chronic disease management. This review aims to examine the mechanisms underlying liver injury and evaluate the potential of plant metabolites in the mitigating and managing various liver diseases.

## 2 Liver anatomy

The liver is the largest internal organ in the body. It is a reddish-brown organ with a roughly cone or wedge-shaped structure. Its narrower end is positioned near the spleen and stomach, while its broader end lies above the small intestine. In humans, the liver accounts for about 2–3 % of total body weight, typically weighing around 1800 g in men and 1400 g in women. Located beneath the right hemidiaphragm in the upper right quadrant of the abdomen, the liver is well-protected by the rib cage. The liver is fully enclosed by the Glisson capsule, except for the area in direct contact with the diaphragm [146].

**Fig. 1:**
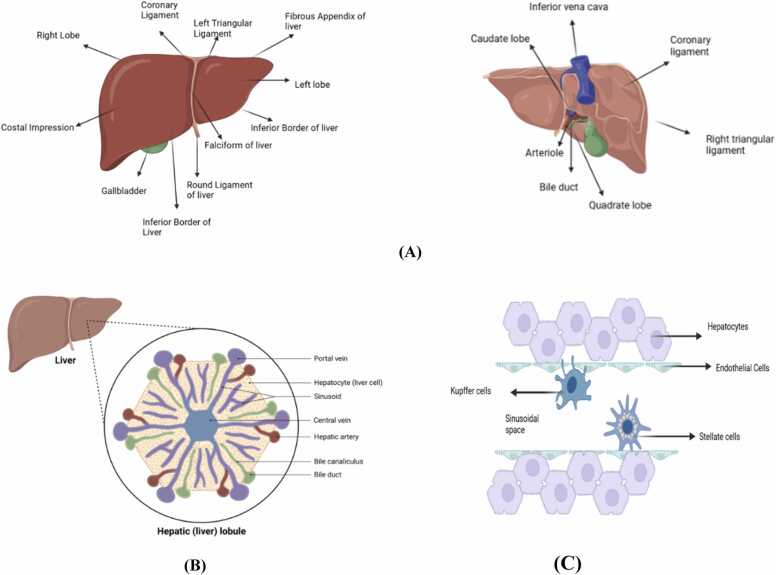
**(A)** Anatomical structure of the liver, showing its lobes, ligaments, and the porta hepatis, with the gallbladder located on the inferior surface **(B)** Microscopic structure of the hepatic lobule, highlighting the portal triad (portal vein, hepatic artery, and bile duct), sinusoids, and central vein **(C)** Cellular composition of the liver, featuring hepatocytes, sinusoidal endothelial cells, Kupffer cells, and stellate cells, which perform essential metabolic, immune, and fibrotic functions.

The liver consists of four lobes: the right, left, caudate, and quadrate lobes, as shown in Fig. 1(A). The falciform ligament, which is sickle-shaped, anchors the liver to the abdominal wall and separates the right and left lobes. These lobes are further divided into eight segments, each consisting of thousands of microscopic lobules. Each lobule contains a duct that channels bile toward the common hepatic duct which drains bile from the liver [15].

From the right lobe, two accessory lobes originate: the quadrate lobe, located on the lower part of the visceral surface, and the caudate lobe, positioned on the upper part of the visceral surface. The porta hepatis, a transverse deep fissure, separates these lobes [19].

At the microscopic level, the liver is organized into lobules, which serve as the fundamental structural units of the organ. Each lobule has a hexagonal structure with a central vein at the center and a portal triad at each corner. The portal triad consists of:

• An arteriole from the hepatic artery supplying oxygenated blood.
• A venule from the hepatic portal vein delivering nutrient-rich blood.
• A bile duct transporting bile from the liver [127], shown in Fig. 1(B).

The liver’s dual blood supply comes from the hepatic artery (25 %) and the hepatic portal vein (75 %), ensuring oxygen and nutrient delivery. Venous drainage occurs through the hepatic veins, which drain into the inferior vena cava. The liver is innervated by the hepatic plexus, composed of sympathetic fibers from the celiac plexus and parasympathetic fibers from the vagus nerve. Glisson’s capsule, a fibrous layer, encases the liver and is innervated by branches of the lower intercostal nerves [97].

## 3 Liver cell types and their functions

The liver is composed of specialized cells that work together to maintain its diverse functions. These include hepatocytes, sinusoidal endothelial cells, Kupffer cells, and stellate cells, as illustrated in Fig. 1(C).

### 3.1 Hepatocytes and endothelial cells

Hepatocytes make up about 60 % of the liver’s cells and account for approximately 80 % of its total mass. They play a pivotal role in synthesizing serum proteins (albumin, coagulation factors), producing bile, and metabolizing lipids, carbohydrates, and proteins. Hepatocytes detoxify various substances, including xenobiotics and steroid hormones, through monooxygenase enzyme systems (Tutty et al., 2022and [157]). These large cells, rich in organelles such as mitochondria, endoplasmic reticulum, lysosomes, and peroxisomes, are arranged in single-cell-thick plates that interact with blood flowing through the space of Disse ( [41]).

Sinusoidal endothelial cells line the hepatic sinusoids and facilitate filtration between the blood and hepatocytes. Their fenestrations allow the exchange of molecules while their strong endocytic capacity aids in absorbing immune complexes and extracellular matrix components[71]. These cells also secrete cytokines and eicosanoids and function as antigen-presenting cells; [58].

### 3.2 Kupffer and stellate cells

Kupffer cells, the largest population of tissue-resident macrophages, reside within the sinusoids and maintain continuous interaction with gut-derived particles [43]. Upon activation, they release inflammatory mediators such as cytokines, reactive oxygen species (ROS), and nitric oxide (NO), contributing to immune responses( [85]). Kupffer cells are highly phagocytic and contain elevated levels of enzymes like glucose 6-phosphate dehydrogenase and peroxidase, enhancing their capacity to clear pathogens [27]

Stellate cells, located in the space of Disse, are the primary storage site for vitamin A, storing retinol and retinyl palmitate within cytoplasmic lipid droplets[28]. Under normal conditions, they regulate extracellular matrix turnover and sinusoidal contractility. However, during liver injury or stress, stellate cells undergo activation, transforming into myofibroblast-like cells that produce extracellular matrix components such as collagen I and fibronectin[135]. This activation is driven by Transforming Growth Factor-beta (TGF-β) and oxidative stress-induced lipid peroxidation( [99]).

Due to its important and central role in the metabolism of xenobiotics, the liver is particularly vulnerable to toxicity. Exposure to xenobiotics can damage liver cells and impair its function. After the liver is exposed to alcohol, drugs, and pollutants, it begins to progress toward damage, resulting in conditions such as hepatosteatosis, cirrhosis and fibrosis. This exposure causes hepatocyte death, resulting in altered levels of various liver enzymes and metabolites, which serve as indicators of liver dysfunction [17]. The reported prime candidate markers in the line-up metabolism of bile when hepatocytes are damaged are transaminases and glutathione [143].

## 4 Generation of hepatotoxicity

**Fig. 2:**
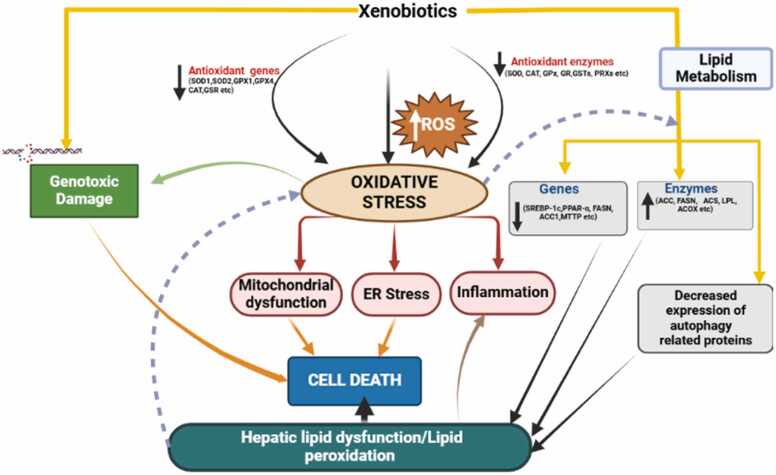
This figure depicts how xenobiotics generate ROS, causing oxidative stress that downregulates antioxidant genes(SOD1, SOD2, GPx, GR, CAT, etc) and enzymes (SOD, CAT, GPx, GR, GSTs, PRX6, etc.) leading to mitochondrial dysfunction, ER stress, and inflammation. These processes result in cell death and hepatic lipid dysfunction. Oxidative stress also triggers genotoxic damage and alters lipid metabolism by downregulating key genes, enzymes(SREBP-1, PPAR-α, FASN, ACC1, ATP, etc.) and enzymes (ACC, FASN, ACS, LPL, ACAT, etc), and autophagy-related proteins, exacerbating liver injury.

**Fig. 3:**
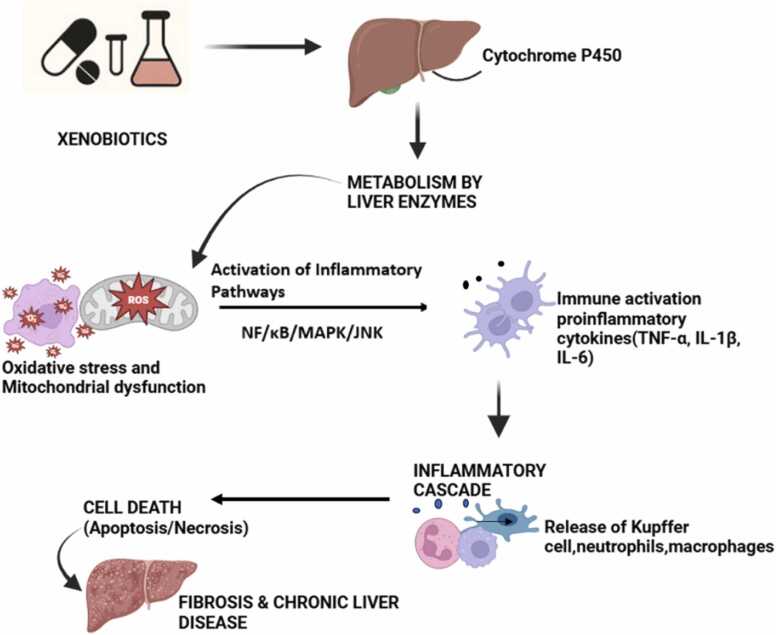
Xenobiotics are metabolized by liver enzymes, primarily cytochrome P450, generating reactive oxygen species (ROS) that cause oxidative stress and mitochondrial dysfunction. This activates inflammatory pathways (NF-κB, MAPK, JNK), leading to the release of proinflammatory cytokines (TNF-α, IL-1β, IL-6) and immune cell recruitment. The resulting inflammatory cascade promotes hepatocyte apoptosis/necrosis, contributing to fibrosis and chronic liver disease.

Hepatotoxicity refers to liver injury resulting from exposure to harmful agents like drugs, environmental toxins, alcohol, or infectious pathogens, as outlined in Table 1. This condition arises through multiple mechanisms, such as oxidative stress, inflammation, and mitochondrial dysfunction, and also disruption of cellular functions [130] as represented in Fig. 2. These processes can result in hepatocyte apoptosis, necrosis, or steatosis, ultimately compromising liver function. Key factors involved in liver damage include ROS, pro-inflammatory cytokines, and the activation of pathways like cytochrome P450, which metabolizes toxic substances into reactive intermediates as demonstrated in Fig. 3 [86].

**Table 1:** Examples of some substances that cause hepatotoxicity.

**S. No**	**Substances**	**Mechanism of Action**	**How It Causes Hepatotoxicity**	**Referenes**
	**Acetaminophen (Paracetamol)**			
	Bioactivation by CYP450 enzymes (mainly CYP2E1) to form NAPQI, a highly reactive intermediate			
	NAPQI binds covalently to cellular proteins, depleting glutathione, leading to oxidative stress, mitochondrial dysfunction, and hepatocyte necrosis	Jaeschke et al., 2024		
	**Bisphenol A (BPA)**			
	Increases ROS production, disrupts calcium homeostasis, and interferes with mitochondrial membrane potential			
	Induces oxidative stress and DNA damage, triggering lipid peroxidation, apoptosis, and inflammatory responses in hepatocytes	[103]		
	**Alcohol (Ethanol)**			
	Induction of CYP2E1 and ADH (alcohol dehydrogenase) increases acetaldehyde levels; acetaldehyde forms protein adducts	Acetaldehyde forms adducts with proteins, triggering immune responses and oxidative stress; ROS production damages membranes and leads to steatosis, hepatitis, and fibrosis		
	[67]			
	**Aflatoxins**			
	Bioactivation by CYP450 enzymes to AFB1–8,9-epoxide, a highly reactive molecule that forms adducts with DNA	Causes mutations in tumor suppressor genes (e.g., TP53), leading to carcinogenesis; oxidative stress also induces apoptosis and necrosis	[2]	
	**Carbon Tetrachloride (CCl₄)**			
	Bioactivation by CYP2E1 and CYP2B1 to trichloromethyl radical (CCl3•), which forms lipid peroxides	Causes lipid peroxidation, protein cross-linking, membrane damage, mitochondrial dysfunction, and necrosis of hepatocytes		
[50]				
	**Iron (Fe) Overload**			
	Iron catalyzes the formation of hydroxyl radicals through the Fenton reaction, leading to oxidative damage	Excess iron accumulates in hepatocytes, causing lipid peroxidation, mitochondrial damage, fibrosis, and cirrhosis	[91]	
	**Methotrexate**			
	Inhibition of dihydrofolate reductase, leading to impaired DNA and RNA synthesis; mitochondrial damage	Disrupts nucleotide synthesis, induces mitochondrial dysfunction, oxidative stress, apoptosis, and fibrosis in hepatocytes	[6]	
	**Amiodarone**			
	Inhibits mitochondrial beta-oxidation of fatty acids, leading to lipid accumulation in hepatocytes	Causes microvesicular steatosis, apoptosis, and necrosis due to impaired energy production and oxidative stress	[39]	
	**Statins**			
	Inhibition of HMG-CoA reductase decreases cholesterol synthesis but also impairs Coenzyme Q10 production	Reduces mitochondrial electron transport chain efficiency, leading to oxidative stress, hepatocyte apoptosis, and mitochondrial dysfunction	[108]	
	**Diclofenac**			
	Bioactivation by CYP2C9 and CYP3A4 to form reactive quinone imine intermediates	Binds covalently to liver proteins, inducing oxidative stress, mitochondrial dysfunction, and inflammatory responses (TNF-α, IL−1β)		
	[114]			
	**Azathioprine**			
	Metabolized to 6-thioguanine nucleotides, which get incorporated into DNA and RNA, impairing synthesis	Disrupts DNA replication, causing mitochondrial dysfunction, oxidative stress, and hepatocyte apoptosis	[136]	
	**Ketoconazole**			
	Inhibits mitochondrial respiratory chain complex I, reducing ATP production	Leads to oxidative stress, impaired fatty acid oxidation, steatosis, and apoptosis of hepatocytes	[124]	
	**Nimesulide**			
	Uncouples oxidative phosphorylation, inhibiting mitochondrial ATP synthesis			
	Causes oxidative stress, hepatocyte necrosis, cholestasis, and hepatocellular damage	[156]		
	**Arsenic**			
	Inhibits mitochondrial respiration by binding to sulfhydryl groups, impairing ATP synthesis	Induces oxidative stress, lipid peroxidation, and DNA damage, causing hepatocyte apoptosis and necrosis	[165]	
	**Tetracycline**			
	Disrupts mitochondrial protein synthesis and induces oxidative stress			
	Causes microvesicular steatosis, mitochondrial dysfunction, and hepatocyte necrosis	[39]		
	**Doxorubicin**			
	Forms semiquinone free radicals via mitochondrial redox cycling, damaging DNA			
	Causes oxidative stress, lipid peroxidation, mitochondrial damage, and hepatocyte apoptosis	[123]		
	**Rifampicin**			
	Bioactivation to reactive metabolites by CYP3A4 and induces ROS production	Causes immune-mediated injury and cholestatic liver damage through oxidative stress and inflammation	[78]	
	**Pyrazinamide**			
	Metabolized to pyrazinoic acid, which inhibits uric acid excretion, causing mitochondrial damage	Leads to oxidative stress, fatty liver, and mitochondrial dysfunction	[111]	
	**Microcystins**			
	Inhibits protein phosphatases PP1 and PP2A, leading to hyperphosphorylation of cytoskeletal proteins	Causes disruption of hepatocyte structure, oxidative stress, and massive hepatic necrosis	[12]

### 4.1 Drug-induced liver injury (DILI)

Hepatotoxicity is the term used to describe liver damage caused by chemicals. Certain medicinal substances, when taken in excessive doses or even within therapeutic limits, can be harmful to the liver. Additionally, other chemical agents, including those used in industries and laboratories, natural toxins such as, herbal remedies and microcystins can also lead to hepatotoxicity [130]. DILI, also known as drug-induced hepatotoxicity, is an acute or chronic response to a natural or synthetic compound. Classification of DILI is based on its clinical presentation (cholestatic, hepatocellular, or mixed), the underlying mechanism of liver damage, or the histological findings observed in a liver biopsy [53]. It is difficult to estimate true incidence of liver disease, yet it has emerged as the leading cause of acute liver failure (ALF) in the United States. Some substances which cause hepatotoxicity are discussed below:

#### 4.1.1 Acetaminophen

Acetaminophen (APAP), also known as paracetamol, was first synthesized in the 1800s and was introduced to the market approximately 65 years ago. From that time it has grown to be one of the most widely used medications globally [101]. A single dose of acetaminophen of less than 7.5–10 g for an adult or 150 mg/kg for a child is unlikely to cause toxicity. A single dose of more than 250 mg/kg or more than 12 g in a 24-hour period might cause toxicity [82]. It is a major cause of acute liver failure. Early research into its toxicity mechanisms revealed that liver enzyme cytochrome P450 catalyze the formation of a reactive metabolite, which depletes glutathione and forms covalent bonds with proteins. Evidence indicates that an acetaminophen overdose can cause mitochondrial dysfunction, either by covalently binding to mitochondrial proteins or through alternative mechanisms [101]. Binding to mitochondrial proteins, especially in the context of glutathione (GSH) depletion, is critical because it disrupts the natural antioxidant functions of mitochondria and alters the ATP-synthase α-subunit, impairing ATP production [93]. During an overdose of acetaminophen intake, glucuronyltransferases and sulfotransferases become saturated, leading the metabolization of drug by cytochrome P450. This process leads to the generation of N-acetyl-p-benzoquinone imine (NAPQI) in amounts that depletes glutathione. If glutathione is not replenished, NAPQI accumulates in hepatocytes, leading to further liver damage [100]. By forming covalent bonds, NAPQI alter the structure and function of cellular proteins.

This disruption increases cytosolic calcium levels and reduction in calcium ATPase activity. Disruption of calcium homeostasis can impair cellular integrity by altering membrane permeability, hence leads to the formation of blebs on the cell membrane and weakening its structural stability [119]. Furthermore, the liver's innate immune system has been shown to play a crucial role in the progression of liver injury during acetaminophen-induced hepatotoxicity [158].

#### 4.1.2 Bisphenol A

Bisphenol A (BPA), chemically known as 2,2-bis(4-hydroxyphenyl)propane, is a widely used synthetic organic compound essential for the manufacture of polycarbonate plastics and epoxy resins[1]. BPA is classified as an endocrine disruptor because it can mimic the actions of natural hormones [64]. Sources of BPA exposure vary depending on environmental, social, and age-related factors. Common sources include baby bottles, beverage containers, food cans, and medical equipment such as polycarbonate hemodialysis devices. The consumption of food and beverages contaminated with BPA can pose significant health risks, particularly the liver being more susceptible[1]. The liver is essential organ for metabolizing and excreting ingested BPA. It was once thought that unconjugated BPA (the active form), when consumed orally, is rapidly conjugated in the liver and subsequently excreted through bile or urine[44]. BPA can be deconjugated by β-glucuronidase enzyme which is present in various tissues, potentially leading to its bioaccumulation in the body [171]. Recent research revealed that the majority of plasma BPA binds to serum proteins, and its accumulation in fat tissue is approximately three times higher than in other organs [34]. BPA has a significant impact on liver enzyme activity, potentially priming the liver for damage. A single injection of 1.2 mg/kg body weight/day of BPA significantly elevated Aspartate Aminotransferase (AST) and Alanine Aminotransferase (ALT) levels within 24 hours in specific pathogen-free C57BL/6 male mice, compared to the control group[1]. Aydin et al. reported that administering 10 and 50 mg/kg of BPA once daily for up to 4 weeks to albino rats led to significant increases in serum Alkaline Phosphatase (ALP), ALT, and bilirubin levels compared to the control group. Similarly, Zaulet and his colleagues found that CD-1 mice treated orally with 200 mg/kg body weight of BPA daily for just 10 days exhibited significant necrotic changes in hepatocytes. The BPA-treated group also showed signs of inflammatory cell infiltration and vascular congestion compared to control [166]. Growing evidence suggests that exposure of BPA causes damage to liver through oxidative stress. Despite the presence of antioxidant system in liver, BPA still induces significant hepatocellular injury. Pirozzi et al., [121]. Studies have demonstrated that BPA leads to a reduction in ATP synthesis in the mitochondria of insulinoma cells in rats [72].

#### 4.1.3 Aflatoxin

Aflatoxins consists of a group of mycotoxins which are carcinogenic and are produced by Aspergillus fungi, which are known to contaminate a substantial portion of the global food supply [37]. Aflatoxins, produced by *Aspergillus flavus* and *Aspergillus parasiticus*, are polysubstituted difuranocoumarin compounds known for their teratogenic, mutagenic, and immunosuppressive effects on both laboratory and farm animals.These toxins leads to severe hepatotoxicity because it primarily affect the liver. Four aflatoxin compounds are produced by *Aspergillus flavus*: aflatoxin B1 (AFB1), aflatoxin B2 (AFB2), aflatoxin G1 (AFG1), and aflatoxin G2 (AFG2). Among all these, AFB1 is the most potent and is extensively documented for its role in the development of hepatocellular carcinoma (HCC) in both humans and animals. The activation of AFB1 to its highly toxic form, AFB1–8,9-epoxide (AFBO), in humans requires Cytochrome P450 1A2 (CYP1A2) and Cytochrome P450 3A4(CYP3A4) which are isoenzymes of hepatic microsomal monooxygenases [26]. AFB1 is also metabolized into various intermediates, including aflatoxicol (AFL), AFB2a, AFQ1 (a major product of CYP3A4 enzymatic activity), and Aflatoxin M1 (AFM1), which is produced by CYP1A2, among others. Interestingly, AFL can also be reconverted into AFB1, hence acting as a reservoir that prolongs its toxic effects [139]. Numerous studies have characterized the oxidative damage induced by AFB1 and its role in hepatotoxicity. AFB1 disrupts the balance between prooxidants and antioxidants, leading to an increase in lipid peroxidation, which damages biological molecules like proteins, lipids and DNA within cellular systems. This cascade of effects can initiate programmed cell death or induce significant genetic alterations in cells, with the severity of the damage being influenced by the dose and duration of AFB1 exposure [159].

#### 4.1.4 Microcystins

Microcystins, including microcystin-leucine arginine (MC-LR), are some of the most toxic and prevalent cyanotoxins produced by cyanobacteria in both freshwater and saltwater algal blooms globally [169]. Acute and chronic exposure to microcystins primarily results in hepatotoxicity, causing cellular damage and genotoxicity in mammalian livers. Acute exposure can lead to hepatomegaly, hemorrhage, and even death in humans or animals, while prolonged exposure may cause chronic liver damage and inflammation. Microcystins primarily activate signaling pathways such as the ERK/JNK/p38 MAPK and IL-6-STAT3 pathways, which trigger oxidative stress and could potentially contribute to carcinogenesis [87]. Moreover, microcystins can synergize with other pollutants, amplifying their toxic effects.

They inhibit a group of enzymes called protein phosphatases, which are responsible for removing phosphate groups from proteins which is a crucial step in various biochemical processes. This inhibition leads to the accumulation of phosphorylated proteins, which is thought to be a mechanism through which microcystins induce liver damage. Hepatocytes from animals treated with microcystins undergo programmed cell death, or apoptosis, leading to liver cell destruction [69].

#### 4.1.5 Metal toxicity

Heavy metals with high atomic weight, such as arsenic (As), cadmium (Cd), chromium (Cr), copper (Cu), lead (Pb), and mercury (Hg) tend to accumulate in the food chain and can harm living organisms at low concentrations [16]. Microcystins enters the human body through various routes like ingestion, inhalation, or skin absorption, and results in severe toxicity. Occupational or environmental exposure to various heavy metals results in various severe effects on health, with oxidative stress being a common mechanism responsible for their toxicity and carcinogenicity, leading to hepatic damage [106]. Moreover, oxidative stress induced by heavy metal exposure activates the nuclear factor (erythroid-derived 2)-like 2/Kelch-like ECH-associated protein 1/antioxidant response elements (Nrf2/Keap1/ARE) pathway is activated by heavy metal induced oxidative stress. Copper and cromium undergo redox-cycling reactions, the primary mechanisms of toxicity for arsenic, cadmium, lead, and mercury involve the reduction of GSH and results in the binding of these metals to the sulfhydryl groups of proteins. This binding disrupts protein function, contributing to cellular damage and dysfunction [68].

#### 4.1.6 Alcohol

Chronic alcohol consumption leads to liver damage through mechanisms involving oxidative stress, release of inflammatory cytokines and the acetaldehyde production [150]. Ethanol metabolism in hepatocytes generates acetaldehyde, a toxic metabolite that damages cellular proteins and DNA. Alcohol consumption also activates Kupffer cells, prompting the release of pro-inflammatory cytokines that intensify hepatocyte injury and contribute to the progression of fibrogenesis [3]. Studies indicate that alcohol-induced oxidative stress plays an important role in liver damage, mainly through activation of Nicotinamide Adenine Dinucleotide Phosphate (NADPH) oxidase and cytochrome P450 2E1 (CYP2E1), which leads to an overproduction of reactive oxygen species (ROS) [35]. Additionally, alcohol-induced changes in gut microbiota contribute to liver injury by releasing endotoxins, which further activate liver immune responses [25].

#### 4.1.7 Hepatitis B and C

Hepatitis B and C viruses causes liver damage primarily through immune-mediated responses. Hepatitis B mechanism involves cytotoxic T lymphocytes targeting Hepatitis B Virus (HBV) infected hepatocytes, leading to inflammation, apoptosis, and fibrosis as the immune system attempts to clear the virus [172]. Hepatitis C Virus (HCV), an RNA virus, replicates in hepatocytes and triggers chronic inflammation, which results in fibrosis and cirrhosis if unresolved [122]. HBV persists in infected hepatocytes within the cell nucleus by forming covalently closed circular DNA (cccDNA), making complete viral eradication challenging [174]. Treatments target viral replication, but the inflammation-driven fibrosis persists. HCV, in contrast, produces various viral proteins, such as Non-structural Protein 5 A (NS5A), that disrupt host lipid metabolism and antioxidant systems, leading to oxidative stress and further liver damage. Chronic HCV can lead to progressive fibrosis due to direct cytopathic effects and immune activation [134].

#### 4.1.8 Non-alcoholic fatty liver disease (NAFLD) and non-alcoholic steatohepatitis (NASH)

NAFLD is primarily defined by the accumulation of lipid droplets in hepatocytes (steatosis), typically resulting from insulin resistance and metabolic syndrome. This buildup triggers hepatocyte stress, lipid peroxidation, and inflammation, which can advance to NASH [38]. Studies indicate that NAFLD pathogenesis is closely associated with insulin resistance, that leads to increase in free fatty acid flux to the liver, oxidative stress, and inflammatory signaling through toll-like receptors (TLRs) [175].

NASH, an advanced form of NAFLD, involves inflammation and cellular damage due to excessive lipid accumulation, mitochondrial dysfunction, and oxidative stress. Persistent hepatocyte injury triggers fibrogenesis and can progress to cirrhosis if not controlled [149]. In NASH, the progression from simple steatosis to steatohepatitis follows a 'two-hit' model in which the first hit involves hepatic steatosis, while the second hit is characterized by inflammation and oxidative stress. Elevated levels of Interleukin-6 (IL-6), Interleukin 1 Beta (IL-1β) and Tumor Necrosis Factor-Alpha (TNF-alpha) are significant mediators of inflammation and fibrosis in NASH [120]. Recent studies suggest mitochondrial dysfunction and increased ROS production as central in NASH progression, alongside an altered microbiome that intensifies inflammatory signaling in the liver [110].

The selection of hepatotoxic agents i.e., acetaminophen, bisphenol A (BPA), aflatoxins, microcystins, heavy metals, alcohol, hepatitis B and C, and non-alcoholic fatty liver disease (NAFLD) progressing to non-alcoholic steatohepatitis (NASH)—was based on their global prevalence, clinical relevance, and diverse mechanisms of hepatotoxicity.These agents encompass a wide range of pathways, including oxidative stress (BPA, microcystins), mitochondrial dysfunction (acetaminophen), immune activation (hepatitis B and C), and fibrosis (NASH and alcohol). They also represent diverse sources of exposure, such as environmental and dietary risks (BPA, aflatoxins, heavy metals) and lifestyle factors (alcohol and microcystins), posing significant health threats, particularly in regions with inadequate regulatory control[83].

## 5 Mechanisms of hepatic injury

### 5.1 ROS and oxidative stress

When xenobiotics are metabolized by the liver, they often undergo biotransformation processes that generate reactive intermediates. These intermediates can produce ROS, which, when present in excess, can overwhelm cellular antioxidant defenses, leading to oxidative stress and subsequent liver injury [10]. Free radicals are highly reactive and unstable molecules or atoms characterized by the presence of unpaired electrons. In biological systems, two main types of free radicals are commonly observed: ROS, which are oxygen-derived, and reactive nitrogen species (RNS), which are nitrogen-derived. Reactive oxygen species are categorized into non-radicals, such as hydrogen peroxide (H2O2) and singlet oxygen (1O2), and radicals, including the superoxide anion (O2●−), alkoxyl radical (RO●), hydroxyl radical (•OH), and peroxyl radical (ROO●) [154].Oxidative stress and ROS play a central role in driving the onset and progression of both acute and chronic liver diseases as previously shown in Fig. 2 [10].

### 5.2 ROS and inflamation

ROS act as vital cytotoxic agents and signaling molecules in the development and progression of inflammatory liver diseases. Their production can originate from infiltrating and resident phagocytes, as well as intracellularly within various liver cell types, in response to cytokine stimulation [151]. While ROS can cause cell damage through extensive lipid peroxidation, they are more commonly involved in modulating signal transduction pathways. This occurs through the modulation of redox-sensitive enzymes, mitochondria, and transcription factors. Consequently, ROS can directly impact and regulate both apoptotic and necrotic cell death pathways as shown in Fig. 3. ROS production can promote lipid peroxidation and increase leakage of electrons from both cell membranes and mitochondria. Furthermore, ROS initiate signal transduction pathways, promote cell death in Kupffer cells, hepatic stellate cells and endothelial cells and trigger caspase activation [141].

### 5.3 ROS and calcium signaling in liver injury

Numerous intracellular signaling pathways contribute to ROS-induced liver cell injury, with one of the most critical mechanisms being the ROS-mediated enhancement of Ca²⁺ influx which leads to an elevation in cytoplasmic Ca2 + concentration ([Ca2 +]cyt). This, in turn, causes a rise in organelle Ca2 + concentrations and activates various downstream intracellular signaling pathways [9]. ROS are known to activate several Ca²⁺-permeable channels in the plasma membrane, facilitating increased calcium influx into cells [40]. Among these channels, the transient receptor potential melastatin 2 (TRPM2) channel stands out as particularly significant. This non-selective cation channel mediates the influx of both calcium (Ca²⁺) and sodium (Na⁺) ions into cells [147]. TRPM2 channels have been identified as key players in mediating cell death and injury in various organs and cell types. In the liver, these channels contribute significantly to paracetamol-induced toxicity and liver ischemia/reperfusion injury [98].

### 5.4 ROS and ischemia-reperfusion (IR) injury

**Fig. 4:**
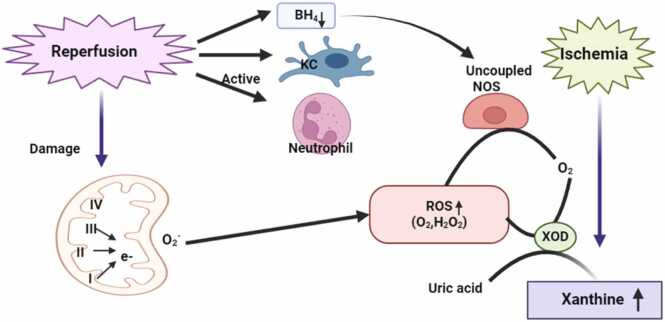
The diagram depicts ischemia-reperfusion (IR)-induced oxidative stress. Ischemia activates uncoupled NOS and xanthine oxidase (XOD), generating reactive oxygen species (ROS). Reperfusion further amplifies ROS through mitochondrial electron transport and neutrophil activation. ROS accumulation leads to oxidative damage, increased uric acid, and tissue injury.

In ischemia-reperfusion (IR) injury, the activation of Kupffer cells and macrophages, combined with mitochondrial dysfunction and the activation of the xanthine/xanthine oxidase system, leads to the production of excessive amount of ROS. These ROS trigger inflammatory responses and apoptosis, causing significant damage to liver tissue [79]. During ischemia-reperfusion (IR) injury, ROS are generated through various pathways, contributing to oxidative stress and cellular damage. Major sources of ROS production include the mitochondrial electron transport chain, where electron leakage results in superoxide anion formation; xanthine oxidase, activated during reperfusion events; NADPH oxidase, which generates ROS in response to cellular signaling; and uncoupled nitric oxide synthase, which shifts from producing nitric oxide to generating superoxide radicals. Tang et al., [151] as shown in Fig. 4.

## 6 Role of plant metabolites in hepatoprotection

Traditional medicines have been utilized for centuries across diverse cultures and regions, with knowledge passed down through generations. They form an integral component of healthcare, particularly in regions with rich biodiversity and ethnomedicinal heritage. Traditional medicinal practices rely on natural resources like plants, minerals, and animal-derived substances, which are believed to possess therapeutic properties (WHO, 2023). The medicinal use of plants in their natural and unprocessed form originated from the observation that certain edible plants could affect specific body functions [45]. For thousands of years, plants have served as a primary source of medicine. These plants act through various mechanisms, including antioxidant activity, enzyme regulation, and immune modulation, contributing to their effectiveness in managing chronic diseases [155]. In recent years, there has been growing interest among researchers and scientists in identifying potential hepatoprotective agents derived from plants to create innovative modern treatments for various liver disorders [163].

### 6.1 Phytochemicals and their classification

**Fig. 5:**
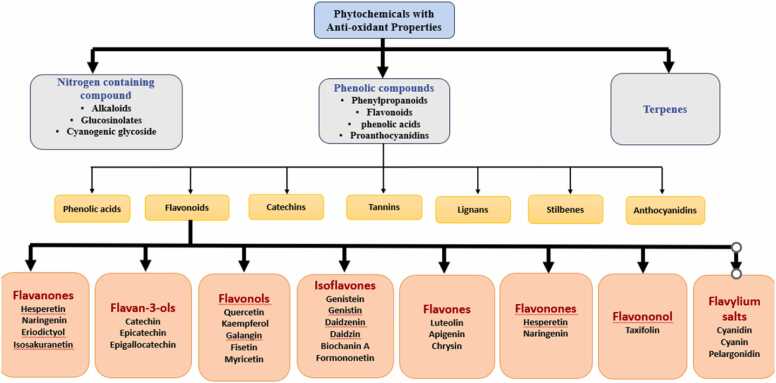
The diagram categorizes phytochemicals with antioxidant properties into three groups: nitrogen-containing compounds (alkaloids, glucosinolates, and cyanogenic glycosides), phenolic compounds (phenylpropanoids, flavonoids, phenolic acids, and proanthocyanidins), and terpenes. Phenolic compounds are further divided into phenolic acids, flavonoids, catechins, tannins, lignans, stilbenes, and anthocyanidins. Flavonoids are classified into flavanones, flavan-3-ols, flavonols, isoflavones, flavones, flavonones, flavonolols, and flavium salts, each with specific bioactive compounds contributing to antioxidant effects.

Phytochemicals which are having antioxidant properties are grouped into three main primary categories according to their biosynthetic pathways, as illustrated in Fig. 5: (a) Nitrogen-containing compounds such as alkaloids, glucosinolates, and cyanogenic glycosides; (b) phenolic compounds like phenylpropanoids and flavonoids; and (c) terpenes [75]. Increasing evidence indicates that dietary phytochemicals have functions beyond their antioxidant properties, affecting various cellular pathways involved in health and disease prevention [113]. Dietary polyphenols are an important category of naturally occurring phytochemicals, including phenolic acids, flavonoids, catechins, tannins, lignans, stilbenes, and anthocyanidins. These compounds are known for their antioxidant, chemopreventive, and diverse pharmacological activities [131]. On the basis of chemical structure flavonoids are divided into various subgroups including flavan-3-ols (such as catechin, epicatechin, and epigallocatechin), isoflavones (like genistein, genistin, daidzein, daidzin, biochanin A, and formononetin), flavones (such as luteolin, apigenin, and chrysin), flavanones (like hesperetin and naringenin), flavonols (such as quercetin, kaempferol, galangin, fisetin, and myricetin), flavononol (like taxifolin), flavylium salts (including cyanidin, cyanin, and pelargonidin), and flavanones (such as hesperetin, naringenin, eriodictyol, and isosakuranetin)[28]. Dietary polyphenols, especially flavonoids, are thought to have strong antioxidant properties that help protect cells from ROS and oxidative stress (OS) playing a crucial role in preventing OS-related diseases and pathological conditions [131]. These compounds reduce inflammation through several mechanisms, including their antioxidant action, interference with oxidative stress signaling, and suppression of pro-inflammatory signaling pathways.

### 6.2 Protective effects and mechanisms of action of bioactive phytochemicals

Numerous in vivo, in vitro, and ex vivo studies have shown that bioactive polyphenols possess a broad spectrum of therapeutic effects, such as immunomodulatory, antimicrobial, antioxidant, antimutagenic, hypolipidemic, hypoglycemic, gastroprotective, anti-inflammatory, anticancer, and chemopreventive properties[88]. Bioactive components help reduce the harmful effects of drugs, chemicals, and their metabolites by modulating intracellular signaling pathways. They activate the Nuclear Factor Erythroid 2-Related Factor 2(Nrf2) molecule through the extracellular-signal-regulated kinase (ERK) and PI3K/Akt pathways, which in turn regulate various transcription factors. However, these components may also exert a negative impact on the SP/Nuclear Receptor 1(SP/NR1) signaling pathways. Overall, bioactive compounds are essential in combating oxidative stress caused by drugs and chemicals. They help normalizing intracellular enzyme levels, safeguarding cells from toxicity, and facilitating the detoxification of harmful compounds within the cell [65].

**Fig. 6:**
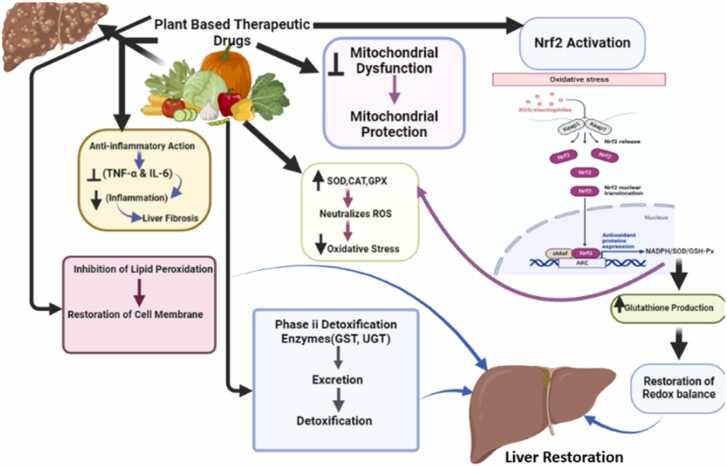
Plant-based therapeutic drugs protect the liver by reducing inflammation (TNF-α, IL-6), inhibiting lipid peroxidation, and restoring cell membranes. They prevent mitochondrial dysfunction by neutralizing ROS via antioxidant enzymes (SOD, CAT, GPX) and activating the Nrf2 pathway, enhancing detoxification (GST, UGT) and glutathione production, leading to liver restoration.

Hepatoprotective herbal drugs protect the liver from harmful effects through various mechanisms, either directly or indirectly acting on hepatocytes. These mechanisms include increasing antioxidant levels or decreasing the formation of ROS, inhibiting cytochrome P450 enzymes, regulating liver enzyme levels, reducing lipid peroxidation, and increasing levels of glutathione or other reducing equivalents. These actions collectively contribute to maintaining proper liver function and protecting it from damage [173] as shown in Fig. 6.

### 6.3 Antioxidant activity

Studies have shown the antioxidant properties of numerous plants, highlighting their potential therapeutic value in preventing and managing diseases related to oxidative stress. Plants produce a range of bioactive compounds, which include phenolics, flavonoids, and tannins, which exhibit strong antioxidant activity [63]. These compounds help neutralize free radicals, thereby mitigating oxidative damage to cellular structures and offering protection against chronic diseases [140]**.** *Andrographis paniculata*, often referred to as the "King of the Bitters," is well-known for its potent antioxidant and hepatoprotective properties. The ethanol extract from the aerial parts of the plant has been shown to significantly protect the liver. In rats exposed to carbon tetrachloride (CCl4), pretreatment with the plant extract at a dose of 300 mg/kg body weight led to a 75 % recovery of ALT and a 14.5 % recovery of AST hepatic enzyme levels. Additionally, the methanol leaf extract of *Andrographis paniculata* has demonstrated effective scavenging activity against 2,2-diphenyl-2-picrylhydrazyl (DPPH) free radicals, achieving a 66 % reduction at a concentration of 500 μg/mL). In a study conducted with Sprague Dawley rats, oral administration of *Callicarpa nudiflora* at a dose of 450 mg/kg body weight once daily for 14 days, followed by two doses of CCl4 (1 mL/kg body weight), resulted in a notable reduction in liver damage markers, including a 63 % decrease in AST levels and a 40 % decrease in ALT levels. Additionally, *Tinospora cordifolia* has been shown to elevate antioxidant enzymes Superoxide Dismutase, Catalase, Glutathione Peroxidase (SOD, CAT, GPx resp,) and nonenzymatic antioxidants like GSH, while reducing lipid peroxidation (LPO) [13]. *Aegle marmelos* at 25 and 50 mg/kg concentrations also attenuated lipid peroxidation (LPO) and xanthine oxidase (XO) activity in a mice model. *Berberis vulgaris* and *Caesalpinia bonducella* enhanced GPx and SOD activities, reduced nitric oxide (NO) levels, lowered lipid peroxidation, and increased non-enzymatic antioxidants (GSH) and antioxidant enzymes (CAT and SOD) levels [73]. Furthermore, *Artemisia annua* L. demonstrated the ability to reduce DNA damage and lipid peroxidation [30].

### 6.4 Anti-inflammatory effects

Liver inflammation is a condition in which the liver tissues continuously react, whether acutely or chronically, to both external and internal factors that compromise the liver's health [152]. During this process, macrophages are activated, which in turn recruit other inflammatory mediators, such as interleukins and TNF-α. This cascade of events amplifies the inflammatory response, eventually contributing to complex conditions that can lead to degenerative diseases, including severe cirrhosis and hepatic carcinoma [96].

Silymarin has demonstrated anti-inflammatory properties in various liver damage models. In rats with alcoholic fatty liver, it exerts its effects by reducing the expression of key inflammatory markers, such as nuclear factor kappa B (NF-κB), IL-6, matrix metalloproteinases (MMP-2 and MMP-13), TGF-β1, the tumor-suppressor Krueppel-like factor, collagen α1, and platelet-derived growth factor (PDGF) signaling. These outcomes were observed in animal models of hepatotoxicity [116]. Phenolic acids, including caffeic acid and ferulic acid, also exhibit strong anti-inflammatory effects by suppressing NF-κB signaling, a key pathway often activated during liver inflammation [132]. By downregulating NF-κB, compounds like caffeic acid reduce the expression of inflammation-related genes, providing protection to the liver against conditions such as NASH and ALD. Studies have indicated that caffeic acid effectively inhibits hepatic lipid peroxidation, which reduces oxidative stress and fat accumulation in the liver [48]. Plants such as *Curcuma longa* (curcumin) and *Rosmarinus officinalis* (carnosol) also modulate inflammatory pathways by targeting Cyclooxygenase-2(COX-2) and Inducible Nitric Oxide Synthase (iNOS) enzymes, which are often elevated in toxic liver injuries [70]. Specifically, curcumin has been shown to activate the Nrf2 pathway, enhancing the liver's antioxidant defenses and providing protection against toxin-induced hepatic damage [77]. This effect not only reduces inflammation but also promotes liver cell survival and recovery. Berberine, a compound derived from plants like *Berberis lycium*, inhibits lipid accumulation and inflammation in the liver by modulating the AMP-Activated Protein Kinase (AMPK) pathway, which regulates cellular energy and fat metabolism [102]. It has proven especially effective in managing NAFLD and steatohepatitis, making it a promising candidate for liver protection (Sharma et al., 2024). Saponins, including those found in *Panax ginseng* (ginsenosides) and *Asparagus racemosus*, also demonstrate hepatoprotective effects by reducing inflammatory markers and supporting liver detoxification. Saponins regulate immune cell responses and can inhibit key cytokines, such as TNF-α and IL-1β, which are involved in inflammatory liver diseases [46]. Furthermore, saponins help stabilize cellular membranes in hepatocytes, shielding them from the damaging effects of free radicals and lipid peroxidation [162].

## 7 Role of plant metabolites in mitigating hepatotoxicity

**Fig. 7:**
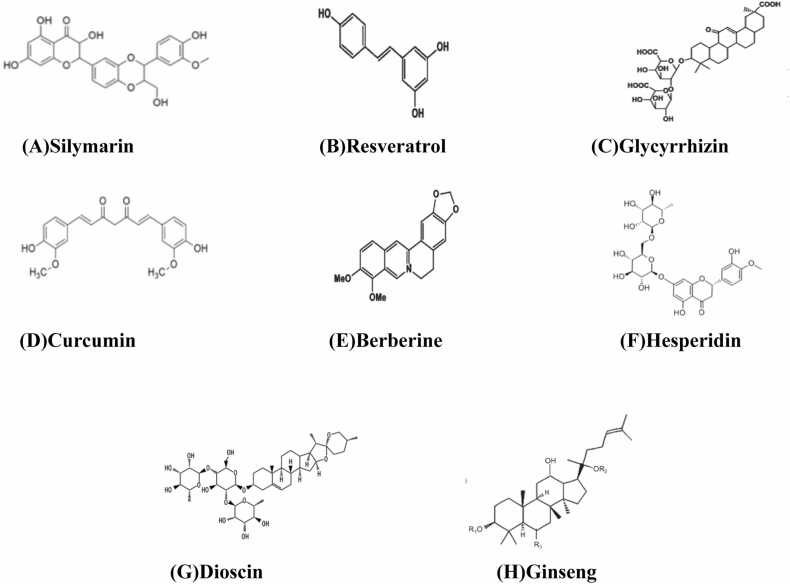
Chemical structures of key plant-derived bioactive compounds with hepatoprotective properties: (A) Silymarin, (B) Resveratrol, (C) Glycyrrhizin, (D) Curcumin, (E) Berberine, (F) Hesperidin, (G) Dioscin, and (H) Ginseng. These compounds exhibit antioxidant, anti-inflammatory, and antifibrotic effects contributing to liver protection and detoxification.

Hepatoprotective agents are therapeutic substances that protect the liver or promote the regeneration of hepatic cells. Certain plants have medicinal properties that help prevent or treat liver disorders. Recently, herbal products have garnered significant attention for managing chronic liver diseases globally, owing to their wide availability, enduring therapeutic effects, and minimal adverse effects [7] as shown in Table 2. Herbal drugs has an important role in regeneration of liver cells, accelerating the healing process, and managing various liver disorders. Numerous traditional plant-based remedies have been tested for their antioxidant properties and hepatoprotective effects against liver damage in experimental animal models [51]. The chemical structure of some of these plant metabolites are shown in Fig. 7 as: (A) Silymarin (B) Resveratrol (C) Glycyrrhizin (D) Curcumin (E) Berberine (F) Hesperidin (G) Dioscin (H) Ginseng and how they are involved in mitigating hepatotoxicity are discussed below:

**Table 2:** Plant metabolites and their hepatoprotective activity.

**S.No**	**Plant Metabolite**	**Plant Source**	**Biological Activity**	**Mechanism of Action**	**References**
	**Silymarin**				
	*Silybum marianum*				
	Hepatoprotective	Antioxidant, reduces lipid peroxidation	[4]		
	**Curcumin**	*Curcuma longa*			
	Hepatoprotective	Anti- inflammatory, inhibits NF-Kb signaling	[32]		
	**Quercetin**	*Allium cepa*, *Camellia sinensis*	Antioxidant,		
Hepatoprotective	Free radical scavenging, reduces oxidative stress	[18]			
	**Berberine**				
	*Berberis lycium*, *Coptis chinensis*	Hepatoprotective	Activates AMPK, improves lipid metabolism	[138]	
	**Andrographolide**				
	*Andrographis paniculata*	Hepatoprotective	Inhibits pro-inflammatory cytokines	[74]	
	**Picroside I & II**				
	*Picrorhiza kurroa*	Hepatoprotective, antioxidant	Protects against oxidative stress-induced damage	Dalavi et al., 2021	
	**Shatavarin IV**				
	*Asparagus racemosus*	Hepatoprotective, antioxidant	Reduces oxidative stress, stabilizes cell membrane		
	[144]				
	**Glycyrrhizin**				
	*Glycyrrhiza glabra*	Hepatoprotective			
	Inhibits HMGB1 release, reduces inflammation	[112]			
	**Genistein**				
	*Glycine max*				
	Antioxidant, hepatoprotective	Reduces lipid accumulation, inhibits oxidative stress	[115]		
	**Rutin**				
	*Ruta graveolens*	Antioxidant, hepatoprotective	Inhibits lipid peroxidation, reduces inflammation	[21]	
	**Epigallocatechin gallate (EGCG)**				
	*Camellia sinensis*	Hepatoprotective	Antioxidant, inhibits fibrosis by reducing TGF-β1	[22]	
	**Baicalin**				
	*Scutellaria baicalensis*	Hepatoprotective, anti-inflammatory	Suppresses inflammatory pathways, reduces apoptosis	[161]	
	**Apigenin**				
	*Petroselinum crispum*	Hepatoprotective, antioxidant	Modulates oxidative stress, reduces inflammation		
	[14]				
	**Diosmin**				
	*Citrus sinensis*, *Citrus aurantium*	Antioxidant, hepatoprotective	Inhibits oxidative damage, reduces pro-inflammatory cytokines	[56]	
	**Catechin**				
	*Vitis vinifera*, *Camellia sinensis*	Hepatoprotective	Reduces lipid peroxidation, enhances antioxidant enzymes	[164]	
	**Rosmarinic acid**				
	*Rosmarinus officinalis*	Hepatoprotective, anti-inflammatory	Inhibits NF-κB activation, reduces oxidative damage	[62]	
	**Thymoquinone**				
	*Nigella sativa*				
	Hepatoprotective, antioxidant	Reduces oxidative stress, anti-inflammatory effects	[52]		
	**Kaempferol**				
	*Allium sativum*	Antioxidant, hepatoprotective	Reduces oxidative stress, inhibits pro-inflammatory enzymes	[24]	
	**Morin**				
	*Morus alba*, *Maclura pomifera*	Hepatoprotective			
	Reduces oxidative stress and apoptosis				
	[145]				
	**Punicalagin**				
	*Punica granatum*	Hepatoprotective, antioxidant	Inhibits oxidative stress, reduces lipid peroxidation	[9]	
	**Apigenin**				
	*Thymus vulgaris*	Antioxidant, hepatoprotective	Modulates inflammation, reduces oxidative stress	[81]	
	**Luteolin**				
	*Capsicum annuum*, *Citrus spp.*	Antioxidant, anti-inflammatory	Inhibits oxidative stress and pro-inflammatory cytokines	[137]	
	**Resveratrol**				
	*Vitis vinifera*	Hepatoprotective, anti-inflammatory	Reduces oxidative stress, inhibits NF-κB activation	[104]	
	**Betulinic acid**				
	*Betula alba*				
	Hepatoprotective, antioxidant	Enhances antioxidant enzymes, reduces oxidative stress	[69]		
	**Silibinin**				
	*Silybum marianum*	Hepatoprotective, antifibrotic	Inhibits fibrogenesis, reduces liver enzymes	[76]	
					
	**Schisandrin B**				
	*Schisandra chinensis*	Hepatoprotective, antioxidant	Increases glutathione levels, reduces lipid peroxidation	[142]	
	**Aucubin**				
	*Aucuba japonica*	Hepatoprotective, anti-inflammatory			
	Suppresses pro-inflammatory cytokines, reduces lipid peroxidation				
	**Chlorogenic acid**				
	*Coffea arabica*, *Helianthus tuberosus*	Antioxidant, hepatoprotective	Inhibits lipid peroxidation, reduces oxidative stress	[95]	
	**Esculetin**				
	*Cichorium intybus*	Hepatoprotective, antioxidant	Inhibits oxidative stress, reduces liver enzyme levels	Aisa, 2020	
	**Rosmarinic acid**				
	*Rosmarinus officinalis*				
	Hepatoprotective, anti-inflammatory	Inhibits NF-κB activation, reduces oxidative damage	[94]

### 7.1 Silymarin

Silymarin, an active compound derived from *Silybum marianum* (L.) Gaertn., commonly known as "milk thistle," is among the oldest plants used for the treatment of liver diseases [42]. The main components of silymarin are silibinin, silidianin, silicristin, and isosilibinin. Among these, silibinin has been widely used for its hepatoprotective and anticancer properties [23]. A clinical study on the immunomodulatory effects of silymarin in hepatitis-C patients revealed its ability to prevent inflammation in both in vivo and in vitro analyses [49]. It also reduces T-cell production and proliferation by lowering pro-inflammatory cytokines while enhancing anti-inflammatory Interleukin-10 (IL-10) [11]. Silibinin has also been shown to inhibit HCV by suppressing clathrin-dependent trafficking. It promotes the formation of clathrin-coated pits and vesicles in hepatocytes, disrupting the clathrin-associated endocytic pathway and interfering with the absorption and trafficking of transferrin [65]. Silymarin exerts hepatoprotective effects through various mechanisms, with the most common involving the modulation of enzymatic and nonenzymatic liver biochemical markers [55] and the induction of nuclear factor-erythroid 2-related factor 2 (Nrf2) expression [29]. The protective effects of silymarin against oxidative stress are partially attributed to a reduction in intracellular calcium in a model of perfused immobilized hepatocytes, leading to enhanced hepatocyte function [133].

### 7.2 Resveratrol

Resveratrol, also known as "trans-3,5,4′-trihydroxystilbene," is a naturally occurring polyphenol found in plants such as *Vitis labrusca* (grapes), *Vaccinium myrtillus L.* (blueberries), and *Rubus idaeus L*. (raspberries). This compound exhibits potent antioxidant properties and acts as a phytoalexin, which plants produce in response to bacterial and fungal attacks [116]. Resveratrol safeguards the liver by reducing oxidative stress during hepatocyte injury, primarily through the modulation of key nuclear transcription factors like Nrf2 and NF-κB. It reduces the expression of genes like Heme Oxygenase-1(HO-1) and Inducible Nitric Oxide Synthase (iNOS), boosting the body’s free radical scavenging activity and supporting phase 2 detoxifying enzymes [8]. Additionally, resveratrol has been shown to suppress the production of pro-inflammatory cytokines, including IL-2, IL-6, and TNF-α, in models of concanavalin A-induced autoimmune hepatitis [33]. In liver damage caused by high cholesterol levels, resveratrol helps by promoting autophagy and downregulating pro-apoptotic proteins such as Bax, caspase-3, and caspase-8 [170]. Furthermore, resveratrol protects against acetaminophen-induced liver toxicity by upregulating Sirtuin 1 (SIRT1) expression, inhibiting p53 signaling, stimulating hepatic cell proliferation, and enhancing liver regeneration through the elevation of cyclin D1 and Cyclin-Dependent Kinase 4 (Cdk4) levels [60].

### 7.3 Glycyrrhizin

Glycyrrhizin (GL), a triterpenoid glycoside extracted from the root of *Glycyrrhiza glabra* L. (licorice root), has been extensively used in the traditional medicine systems of Nepal, India, China, and other countries for the treatment of jaundice [5]. GL exerts its hepatoprotective effects through different mechanisms, such as acting as an anti-inflammatory agent and enhancing antioxidant defenses in hepatic cells [128]. One of the mechanisms by which GL mediates its anti-inflammatory effect is by reducing the levels of high-mobility group box protein 1 (HMGB1) or disrupting its binding to the promoter region of glutathione S-transferase omega-1 (GSTO1) [116]. Emerging evidence has shown that GL also possesses significant antioxidant activity both in vivo and in vitro. It has been reported that GL protects hepatocytes from oxidative stress induced by azathioprine or t-butyl hydroperoxide (t-BHP), by reducing intracellular ROS formation, GSH depletion, and oxidative damage [90]. In a clinical study involving long-term alcohol consumption, GL effectively reduced serum levels of AST, ALT, and gamma-glutamyl transferase (GGT) in patients [47]. Furthermore, GL has been shown to enhance the accumulation of cisplatin in cancer cells by inhibiting its efflux, thus reversing cisplatin resistance in hepatocellular carcinoma (HCC) [160]. Research by Zhang et al. discovered that GL induces autophagy and inhibits HCC tumor growth in a xenograft model by suppressing the AKT/mTOR and ERK1/2 signaling pathways [168].

### 7.4 Curcumin

Curcumin, the main curcuminoid found in the rhizome of *Curcuma longa* (turmeric), is a chemical compound known as diferuloylmethane. It consists of a diferulic acid moiety linked to a methylene group or other carbon moieties and primarily exists in a keto-enol tautomeric form [116]. Turmeric has a long history of traditional use in the treatment of liver diseases, including bilirubin-related conditions like jaundice and other hepatic complications [163]. The hepatoprotective mechanism of curcumin is thought to arise from its antioxidant properties and its ability to activate phase 2 detoxifying/antioxidant enzymes, such as heme oxygenase-1 (HO-1), NADPH quinone oxidoreductase-1 (NQO1), and the Nrf2/Kelch-like ECH-associated protein 1 (Keap1)/antioxidant-responsive element (ARE) pathway. Recent studies showed that in curcumin-resistant hepatic carcinoma cells, the Chk1-associated G2/M cell cycle block might contribute to curcumin resistance, with Chk1 being a potential target for improving the therapeutic efficacy of curcumin [116]. Curcumin exerts protective and therapeutic effects in liver diseases related to oxidative stress by inhibiting pro-inflammatory cytokines, reducing lipid peroxidation products, suppressing hepatic stellate cell activation, and preventing Akt activation. It also ameliorates oxidative stress by increasing the expression of Nrf2, SOD, CAT, and GSH. Curcumin acts as a free radical scavenger, neutralizing a range of ROS through its active phenolic pharmacophore, β-diketone, and methoxy groups [118]. In cases of aflatoxin B1-induced hepatotoxicity, curcumin has been shown to protect liver cells from lipid peroxidation (LPO) and oxidative DNA damage. Its antioxidant potential helps to restore elevated serum marker enzymes, reduce LPO, increase antioxidant enzyme levels, and lower the excretion of DNA adducts, thereby providing protection against liver toxicity induced by aflatoxin B1 (Dai et al., 2022).

### 7.5 Berberine

Berberine, an isoquinoline alkaloid, is derived from the roots, rhizomes, and stem bark of *Berberis aristata* DC, commonly known as "barberry." It has been traditionally used as a remedy for liver-related ailments [66]. The primary bioactive compounds in *Berberis aristata* include berberine, oxyberberine, berbamine, aromoline, karachine, and oxycanthine, with berberine being the most studied for its hepatoprotective properties [63]. Berberine exerts antioxidant effects, helping to alleviate oxidative stress and reduce apoptosis by enhancing the Bcl-2/Bax ratio in ischemia/reperfusion-induced liver injury, as well as inhibiting caspase-3 cleavage in liver cells. Research also indicates that berberine-loaded nanoparticles (BBR-SLNs) can mitigate hepatosteatosis by decreasing lipogenesis and enhancing lipolysis in hepatocytes. These nanoparticles reduce the expression of lipogenic genes such as stearoyl-CoA desaturase, sterol regulatory element-binding protein 1c, and fatty acid synthase, while increasing lipolytic gene expression, such as carnitine palmitoyltransferase-1 [20]. Furthermore, berberine demonstrates antifibrotic effects through several mechanisms, one of which is the induction of G1 phase arrest in hepatic stellate cells (HSCs), preventing their activation—an essential process in liver fibrosis. Additionally, berberine has been shown to inhibit the phosphorylation of forkhead box O1 (FoxO1) and Akt in rats with bile duct ligation-induced liver fibrosis and in HSCs, further supporting its potential as a treatment for liver fibrosis [173].

### 7.6 Hesperidin

Hesperidin (C28H34O15) is a glycosidic flavanone primarily found in citrus fruits like sweet oranges and lemons, making it a cost-effective by-product of citrus cultivation. Research has extensively explored the radical scavenging properties and antioxidant activity of hesperidin across various assay systems. In particular, its antioxidant effects have been demonstrated in liver homogenates, where it reduced hydroperoxide-induced chemiluminescence [31]. Additionally, hesperidin has shown inhibitory activity on non-enzymatic lipid peroxidation in rat brain mitochondria and in the enzyme 15-lipoxygenase [59]. Hesperidin is also known for its hepatoprotective effects. It boosts the activities of liver enzymes including serum and tissue-specific enzymes and hence protects the liver from doxorubicin-induced hepatotoxicity. It has been found to significantly increase hepatic glutathione levels, and the activities of glutathione peroxidase, glutathione-S-transferase, and peroxidase, while reducing lipid peroxidation [109]. Hesperidin also exhibits anticancer properties against various carcinomas, including those of the colon, esophagus, tongue, and urinary bladder [36]. In studies involving carbon tetrachloride (CCl4), a known hepatotoxin, hesperidin has demonstrated protective effects on the liver. When rats were administered a single dose of CCl4 (2 mL/kg), they experienced increased levels of AST and ALT, a notable reduction in liver glutathione, and decreased activities of antioxidant enzymes such as SOD and GPx. However, treatment with hesperidin (10 and 100 mg/kg orally for 28 days) restored these enzyme activities, indicating its antioxidant properties and hepatoprotective potential [148]. Hesperidin has also been shown to protect against APAP -induced liver damage. In an in vivo study, administration of APAP (100 and 200 mg/kg orally for 14 days) resulted in significant depletion of liver glutathione, impairment of antioxidant enzymes (GST, GR, GPx, and CAT), and increased serum levels of AST, ALT, LDH, blood urea nitrogen, and creatinine. Treatment with hesperidin (750 mg/kg orally for 14 days) effectively reversed these adverse effects, demonstrating its ability to prevent oxidative stress and liver toxicity induced by Acetaminophen (APAP) [129].

### 7.7 Dioscin

Dioscin, a steroidal saponin predominantly found in *Dioscorea opposita*, is renowned for its therapeutic properties, particularly its protective effects against fatty liver disease. Research has shown that dioscin effectively reduces oxidative stress by scavenging reactive oxygen species (ROS) and boosting the activity of key antioxidant enzymes such as SOD, CAT, and glutathione peroxidase (GSH-Px). This antioxidant activity shields hepatocytes from lipid peroxidation and oxidative damage, both of which are common contributors to liver injury [159]. Additionally, dioscin exhibits anti-inflammatory properties by inhibiting inflammatory mediators like TNF-α, IL-6, and cyclooxygenase-2 (COX-2). It also regulates nuclear factor-kappa B (NF-κB) signaling, a key pathway involved in liver inflammation, thereby reducing inflammation-induced liver damage [125]. Recent studies have demonstrated that dioscin helps reduce lipid accumulation in the liver, which enhances its anti-obesity and hepatoprotective effects. It increases energy expenditure, promotes oxygen consumption, and lowers serum liver enzyme levels, such as AST and ALT, which are typically elevated in liver injury [105]. Furthermore, dioscin has been shown to combat oxidative damage, reduce triglyceride and cholesterol synthesis, and inhibit mitogen-activated protein kinase (MAPK) phosphorylation. It also induces β-oxidation of fatty acids, aiding in fat breakdown, and promotes autophagy, a process that clears damaged cellular components. These combined actions make dioscin a promising candidate for the treatment of conditions like obesity and NAFLD [107].

### 7.8 Ginseng

Ginseng, particularly its active constituents known as ginsenosides, has long been celebrated in traditional medicine for its wide-ranging health benefits, including hepatoprotection. Ginseng contains a variety of bioactive compounds, including ginsenosides, fatty acids, polysaccharides, and mineral oils, with its pharmacological effects primarily attributed to the ginsenosides [80]. These ginsenosides are categorized into protopanaxadiol and protopanaxatriol types, each with unique effects on the liver.

Ginseng has been studied for its ability to protect against liver damage induced by various hepatotoxic agents, including drugs, alcohol, contaminants, and poisons. These agents can lead to conditions such as hepatitis, cirrhosis, fibrosis, and hepatocellular carcinoma (HCC). Numerous in vitro and animal studies have shown that ginseng extracts, especially ginsenosides, offer protective effects in liver injury models induced by these toxins [117]. One of the primary ginsenosides, ginsenoside Rg1, has demonstrated potent antioxidant properties, reducing oxidative stress and protecting liver cells from oxidative damage [54]. Additionally, ginsenosides such as Rg1 and Rb1 have been shown to inhibit the activation of hepatic stellate cells, which play a central role in the development of liver fibrosis. These compounds achieve this by downregulating key fibrogenic mediators, including TGF-β, a key driver of fibrogenesis [92]. Furthermore, ginseng exerts protective effects by modulating apoptotic pathways. It inhibits the activation of caspases, which are involved in the execution of apoptosis, while promoting the expression of anti-apoptotic proteins like Bcl-2 [57]. In experimental models of liver fibrosis, ginsenosides have been found to reduce collagen deposition and suppress fibrogenic markers, making ginseng a promising candidate for the prevention and treatment of liver fibrosis [92].

## 8 Conclusion

Natural products have emerged as a highly promising alternative to conventional pharmaceuticals, particularly in the realm of liver protection. Their effectiveness, combined with minimal side effects and the added benefit of being dietary components, positions them as preferable options over synthetic drugs that often pose toxicity risks. Herbal hepatoprotective agents are distinguished by their safety, efficacy, and cost-effectiveness, contributing significantly to the maintenance of liver health and functionality. Despite the availability of numerous herbal formulations, the specific mechanisms by which they exert their protective effects are not yet fully understood. This underscores the pressing need for comprehensive research to elucidate the molecular pathways and interactions that drive their hepatoprotective actions. Our review highlights the critical importance of further investigation into the roles of active natural constituents and their derivatives in addressing liver diseases, thereby advocating for enhanced efforts to initiate human clinical trials to substantiate their therapeutic potential.

## CRediT authorship contribution statement

**Reshi Mohd Salim:** Validation, Supervision, Resources, Methodology, Investigation, Formal analysis, Conceptualization. **Ganie Shahid Yousuf:** Writing – original draft, Methodology, Formal analysis, Conceptualization. **Reyaz Adfar:** Writing – original draft, Methodology, Formal analysis, Conceptualization. **Javaid Darakhshan:** Writing – original draft, Methodology, Formal analysis, Conceptualization. **Qadri Syed Sanober:** Writing – review & editing, Writing – original draft, Methodology, Formal analysis, Data curation, Conceptualization.

## Declaration of Competing Interest

The authors declare that they have no known competing financial interests or personal relationships that could have appeared to influence the work reported in this paper.

## Data Availability

Data will be made available on request.
